# Oxidized Low-Density Lipoprotein Enhances Toll-like Receptor-Mediated Osteoclastogenic Responses in the Absence of Exogenous RANKL: Implications for Inflammatory Osteoclastogenesis in Periodontitis

**DOI:** 10.3390/medicina62081562

**Published:** 2026-08-14

**Authors:** Kimiko Ohgi, Hiroshi Kajiya, Yoshiyuki Nagaoka, Nana Yamamoto, Naoki Maruo, Hiroaki Yamato, Nanako Tsuchimochi, Masanobu Nakagami, Aya Fujioka, Yasunori Yoshinaga

**Affiliations:** 1 Section of Periodontology, Department of Odontology, Fukuoka Dental College, Fukuoka 814-0175, Japan; m19840813@fdcnet.ac.jp (N.Y.); maruo@fdcnet.ac.jp (N.M.); yamato@fdcnet.ac.jp (H.Y.); nanakot@fdcnet.ac.jp (N.T.); nakagami@fdcnet.ac.jp (M.N.); fujiokaa@fdcnet.ac.jp (A.F.); yoshiyasu@fdcnet.ac.jp (Y.Y.); 2 Department of Physiological Science and Molecular Biology, Fukuoka Dental College, Fukuoka 814-0175, Japan; kajiya@fdcnet.ac.jp (H.K.); nagaoka@fdcnet.ac.jp (Y.N.); 3 Oral Medicine Research Center, Fukuoka Dental College, Fukuoka 814-0175, Japan

**Keywords:** chronic inflammation, dyslipidemias, oxidized LDL, LOX-1, Toll-like receptors, osteoclastogenesis, periodontitis

## Abstract

*Background and Objectives:* Oxidized LDL (oxLDL) and Toll-like receptor (TLR) signaling are implicated in inflammatory bone loss and periodontitis, but their cooperative effects on osteoclastogenesis remain unclear. We investigated whether TLR2/4 stimulation regulates the transcription of LOX-1 (lectin-like oxidized low-density lipoprotein receptor-1), a receptor for oxLDL, and whether oxLDL enhances TLR-mediated osteoclastogenic responses in the absence of exogenous RANKL. *Materials and Methods:* Mouse bone marrow cells (BMCs) were differentiated into bone marrow macrophages (BMMs) with M-CSF and stimulated with TLR ligands (Pam3CSK4 for TLR2 and Lipid A for TLR4) and/or oxLDL, with or without exogenous RANKL. Raw264.7 cells were stimulated with TLR ligands or Porphyromonas gingivalis lipopolysaccharide (LPS). LOX-1 transcriptional activity and osteoclastogenic responses were evaluated using luciferase assays, quantitative RT-PCR, and tartrate-resistant acid phosphatase (TRAP) staining. *Results:* In Raw264.7 cells, stimulation with Pam3CSK4, Lipid A, or LPS significantly increased LOX-1 promoter activity. In BMCs, Pam3CSK4 significantly increased LOX-1 and MyD88 mRNA expression on day 1, whereas expression decreased by day 3; these changes were suppressed by a TLR2 inhibitor. Lipid A significantly increased LOX-1 and MyD88 mRNA expression on day 3, and the increase in LOX-1 expression was inhibited by a TLR4 inhibitor. Functionally, Pam3CSK4 or Lipid A alone increased mononuclear TRAP-positive cells but did not induce multinucleated TRAP-positive cells. In contrast, co-stimulation of oxLDL with either Pam3CSK4 or Lipid A promoted multinucleated TRAP-positive cell formation even in the absence of exogenous RANKL. *Conclusions:* TLR2/4 stimulation transiently enhanced LOX-1 transcription and expression in association with increased MyD88 expression. TLR ligands cooperated with oxLDL to promote multinucleated TRAP-positive cell formation in the absence of exogenous RANKL, suggesting that inflammatory and metabolic signals may act synergistically during osteoclastogenesis. These findings improve our understanding of how dyslipidemia-associated factors may contribute to inflammatory bone-resorptive diseases, including periodontitis.

## 1. Introduction

Chronic inflammatory diseases such as periodontitis are characterized by persistent activation of innate immune signaling pathways triggered by bacterial infection. Toll-like receptors (TLRs), particularly TLR2 and TLR4, play central roles in host responses to periodontal pathogens and contribute to inflammatory bone remodeling [1,2,3,4,5,6,7,8,9,10,11,12]. Lipopolysaccharide (LPS), a major component of Gram-negative bacteria, has been reported to stimulate osteoclast-related inflammatory responses through TLR signaling [10,11,12]. However, the effects of TLR2/4 signaling on osteoclastogenesis remain controversial, as both stimulatory and inhibitory effects have been reported depending on experimental conditions [13,14,15,16,17]. The mechanisms regulating TLR-mediated osteoclastogenic responses during chronic inflammation therefore remain incompletely understood.

Dyslipidemia, a major component of metabolic syndrome, has increased worldwide and is increasingly recognized as an important modifier of chronic inflammatory diseases. Beyond its established role in cardiovascular disease, dyslipidemia has also been implicated in skeletal disorders and inflammatory bone loss through the accumulation of oxidized lipoproteins and persistent activation of innate immune pathways [18,19]. These observations suggest that metabolic abnormalities may substantially influence inflammatory responses in periodontal tissues. Elevated circulating low-density lipoprotein (LDL) undergoes oxidative modification to generate oxLDL, a bioactive lipoprotein that amplifies inflammatory responses and tissue injury [20,21,22]. Experimental and clinical evidence suggests an association between hyperlipidemia and increased periodontal tissue destruction accompanied by alveolar bone loss [23,24,25,26,27]. In vitro studies have demonstrated that oxLDL activates inflammatory signaling pathways and enhances the production of pro-inflammatory cytokines in gingival cells [27]. Taken together, the present observations support the concept that disturbances in lipid metabolism may modify inflammatory responses and bone remodeling in periodontal tissues. Increasing evidence indicates that metabolic disorders alter innate immune signaling in multiple inflammatory tissues, suggesting that dyslipidemia may influence periodontal inflammation through mechanisms beyond lipid accumulation.

Lectin-like oxidized low-density lipoprotein receptor-1 (LOX-1) is a major receptor for oxLDL originally identified in endothelial and vascular tissues [28]. In addition to oxLDL, Lox-1 recognizes multiple ligands including bacterial components [29]. Experimental studies have consistently shown that activation of TLR2 signaling upregulates LOX-1 expression in mouse bone marrow macrophages and enhances osteoclastogenic responses [30]. Furthermore, LOX-1 deficiency alters inflammatory bone responses in vivo [31]. Collectively, these observations raise the possibility that LOX-1 and TLR signaling interact during inflammatory bone remodeling, although the molecular mechanisms responsible for this interaction remain poorly understood.

Because TLR stimulation is widely used as an in vitro model of bacterial infection–associated chronic inflammation [32,33], the present study investigated whether TLR2/4 stimulation regulates LOX-1 expression and whether oxLDL cooperates with TLR signaling to promote osteoclastogenic responses in vitro. In addition, we examined changes in MyD88 expression associated with TLR-mediated LOX-1 induction. Elucidating these interactions may improve our understanding of how metabolic dysregulation and innate immune activation cooperate during inflammatory bone remodeling associated with periodontitis.

## 2. Materials and Methods

### 2.1. Cell Preparation for qRT-PCR Experiments

Mouse bone marrow cells (BMCs) were isolated from the tibiae and femora of 4–5-week-old male ddY mice (Japan SLC, Shizuoka, Japan) euthanized by cervical dislocation under isoflurane anesthesia (0.5–5% in oxygen). Cells were cultured in α-minimum essential medium (α-MEM; Invitrogen, Grand Island, NY, USA) supplemented with 10% fetal bovine serum (FBS; Biowest, Nuaille, France), penicillin G (100 U/mL), and streptomycin sulfate (0.15 mg/mL).

After overnight incubation, non-adherent cells were collected and cultured with macrophage colony-stimulating factor (M-CSF; 20 ng/mL; PeproTech, Rocky Hill, NJ, USA) for 3 days to induce differentiation into bone marrow macrophages (BMMs). Cells were then stimulated with Pam3CSK4 (TLR2 ligand; 100 ng/mL; IMGENEX, San Diego, CA, USA) or Lipid A (TLR4 ligand; 100 ng/mL; Avanti Polar Lipids, Alabaster, AL, USA), for the indicated times, with or without pretreatment with TLR2 or TLR4 inhibitors. These BMMs were subsequently subjected to quantitative RT-PCR analysis.

The concentrations of Pam3CSK4 and Lipid A differed among experiments because they were selected based on the specific requirements and sensitivity of each experimental system (e.g., luciferase reporter assays, gene expression analyses, immunocytochemistry, Western blotting, and osteoclastogenic assays).

### 2.2. Cell Preparation for Osteoclastogeneic Assays

Spleen-derived cells were used for osteoclastogenic assays because they provide abundant monocyte/macrophage precursors suitable for multinucleated TRAP-positive cell formation, whereas bone marrow-derived cells were used for gene expression analyses.

Spleens were mechanically dissociated, and the resulting cell suspensions were collected by centrifugation and cultured in α-MEM supplemented with 10% FBS and M-CSF (20 ng/mL). During the first 3 days, the cells were cultured with M-CSF in the presence or absence of Pam3CSK4 (100 ng/mL) or Lipid A (100 ng/mL). On day 3, oxidized low-density lipoprotein (oxLDL; 20 μg/mL; Alfa Aesar, Lancashire, UK) and/or receptor activator of NF-κB ligand (RANKL; 80 ng/mL; PeproTech, Rocky Hill, NJ, USA) were added as indicated, while M-CSF and TLR ligands were maintained throughout the culture period. Cells were cultured for an additional 3 days and then subjected to TRAP staining.

### 2.3. Inhibitor Treatment

For pathway inhibition experiments, BMMs were pretreated for 1 h with a neutralizing anti-LOX-1 antibody (10 μg/mL; AF1564, R&D Systems, Minneapolis, MN, USA), U0126 (MEK inhibitor; 10 μmol/L), SP600125 (JNK inhibitor; 5 μmol/L), or SB203580 (p38 inhibitor; 10 μmol/L), according to a previously published protocol [34] with minor modifications. Cells were subsequently stimulated with Pam3CSK4 or Lipid A as indicated. Control cells received either vehicle (DMSO) or an equivalent volume of antibody diluent, as appropriate.

### 2.4. TRAP Staining

To evaluate osteoclastogenic responses, cells were fixed with 3.7% formaldehyde and stained for tartrate-resistant acid phosphatase (TRAP) using the Acid Phosphatase Leukocyte Kit (Sigma-Aldrich, St. Louis, MO, USA). TRAP-positive mononuclear and multinucleated cells were quantified by blinded counting in randomly selected microscopic fields. Multinucleated TRAP-positive cells were defined as TRAP-positive cells containing three or more nuclei. Mononuclear TRAP-positive cells were defined as TRAP-positive cells containing a single nucleus.

Each condition was tested in triplicate, and all experiments were repeated at least three times.

### 2.5. Transcriptional Activity Assay

Raw264.7 murine macrophages (ATCC, Manassas, VA, USA) were stimulated with lipopolysaccharide (LPS) from Porphyromonas gingivalis (100 ng/mL), Pam3CSK4 (TLR2 ligand; 5 ng/mL), or Lipid A (TLR4 ligand; 0.1 ng/mL) for the indicated times.

For promoter assays, the murine LOX-1 promoter construct (−3000 to +58)/pGL3-basic was kindly provided by Akinobu Ota [34]. Raw264.7 cells were transfected with the construct using Lipofectamine 3000 (Thermo Fisher Scientific, Waltham, MA, USA). Luciferase activity was measured using a luciferase reporter assay system (Promega, Madison, WI, USA). Cells were co-transfected with a pCMV-β-galactosidase reporter plasmid (Promega), and transfection efficiency was normalized by measuring β-galactosidase activity.

### 2.6. Quantitative Reverse Transcription Polymerase Chain Reaction (qRT-PCR)

Total RNA was extracted using TRIzol reagent (Life Technologies Corporation, Rockville, MD, USA). First-strand cDNA was synthesized from 1 μg total RNA using SuperScript II reverse transcriptase according to the manufacturer’s instructions (Invitrogen, Grand Island, NY, USA). Quantitative real-time PCR was performed using Power SYBR Green PCR Master Mix (Applied Biosystems, Foster City, CA, USA) in a CFX96 real-time PCR system (Bio-Rad Laboratories, Hercules, CA, USA). Amplification was performed in a total volume of 20 μL under the following conditions: 95 °C for 30 s, followed by 40 cycles of 95 °C for 5 s, 60 °C for 10 s, and 72 °C for 40 s.

Target gene expression levels were normalized to GAPDH expression and calculated using relative expression units. The following primer sequences were used: LOX-1: sense 5′-CTGCGAATGACGAGCCTGA-3′, antisense 5′-TCACTGACAACACCAGGCAGAG-3′; MyD88: sense 5′-TACAGGTGGCCAGAGTGGAA-3′, antisense 5′-GCAGTAGCAGATAAAGGCATCGAA-3′; GAPDH: sense 5′-TGTGTCCGTCGTGGATCTGA-3′, antisense 5′-TTGCTGTTGAAGTCGCAGGAG-3′.

Relative gene expression levels were calculated using the 2−ΔΔCt method with GAPDH as the internal control. For statistical analyses, ΔCt values were used.

### 2.7. Immunocytochemistry

Cells were fixed with 4% formaldehyde for 5 min and permeabilized with 0.05% Triton X-100 in phosphate-buffered saline (PBS) for 5 min. After blocking with 3% normal goat serum for 40 min at room temperature, cells were incubated overnight at 4 °C with a rabbit polyclonal anti-Lox-1 antibody (1:100 dilution; ab60178, Abcam, Cambridge, UK). After washing three times with PBS, cells were incubated with DyLight 488-conjugated goat anti-rabbit IgG secondary antibody (1:1500 dilution; Vector Laboratories, Burlingame, CA, USA) for 30 min at room temperature. Cell nuclei were counterstained with 4′,6-diamidino-2-phenylindole (DAPI) using mounting medium. Fluorescence images were acquired using a fluorescence microscope (TMD300, Nikon, Tokyo, Japan). For quantitative analysis, Lox-1-positive cells were counted in multiple randomly selected microscopic fields per well by blinded observers, and the mean number of positive cells per well was calculated.

### 2.8. Western Blot Analysis

Raw264.7 cells were stimulated with Pam3CSK4 (5 ng/mL), or Lipid A (0.1 ng/mL) for the indicated times.

Cells were lysed in Tris-NaCl-Tween (TNT) buffer containing 20 mM tris–HCl (pH 7.5), 200 mM NaCl, 1% Triton X-100, 1 mM dithiothreitol (DTT), and protease inhibitors (Roche, Basel, Switzerland). The protein content of the samples was measured using Pierce reagents, following the manufacturer’s protocol. Protein samples (20 μg) were subjected to sodium dodecyl sulfate-polyacrylamide gel electrophoresis, and proteins were transferred to a polyvinylidene difluoride (PVDF) membrane (100 V, 1 h, 4 °C). The membranes were incubated with anti-ERK1/2 antibody (Abcam, Cat. No. ab184699; 1:10,000 dilution) and anti-β-actin antibody (1:1000 dilution) in 5% (*w*/*v*) skim milk solution supplemented with 0.01% (*w*/*v*) sodium azide overnight at 4 °C. The blots were washed in Tris- buffered saline with Tween (TTBS) (10 mM tris–HCl, 50 mM NaCl, 0.25% Tween 20) and incubated with an appropriate secondary antibody for 30 min at room temperature. The immunoreactive proteins were visualized using enhanced chemiluminescence reagents (GE Healthcare, Tokyo, Japan).

### 2.9. Data Analysis

At least three independent experiments were performed, with three replicates per experiment. Data are presented as mean ± SEM. Homogeneity of variance was assessed using Levene’s test. For data with equal variances, statistical significance was evaluated using one-way analysis of variance (ANOVA) followed by Dunnett’s post hoc test. For data with unequal variances, Welch’s ANOVA followed by the Games–Howell post hoc test was used. Statistical analyses were performed using SPSS version 29.0 (IBM Corp., Armonk, NY, USA). A *p*-value < 0.05 was considered statistically significant.

## 3. Results

### 3.1. TLR2/4 Stimulation Transiently Increases LOX-1 Promoter Activity in Raw264.7 Cells

**Figure 1:**
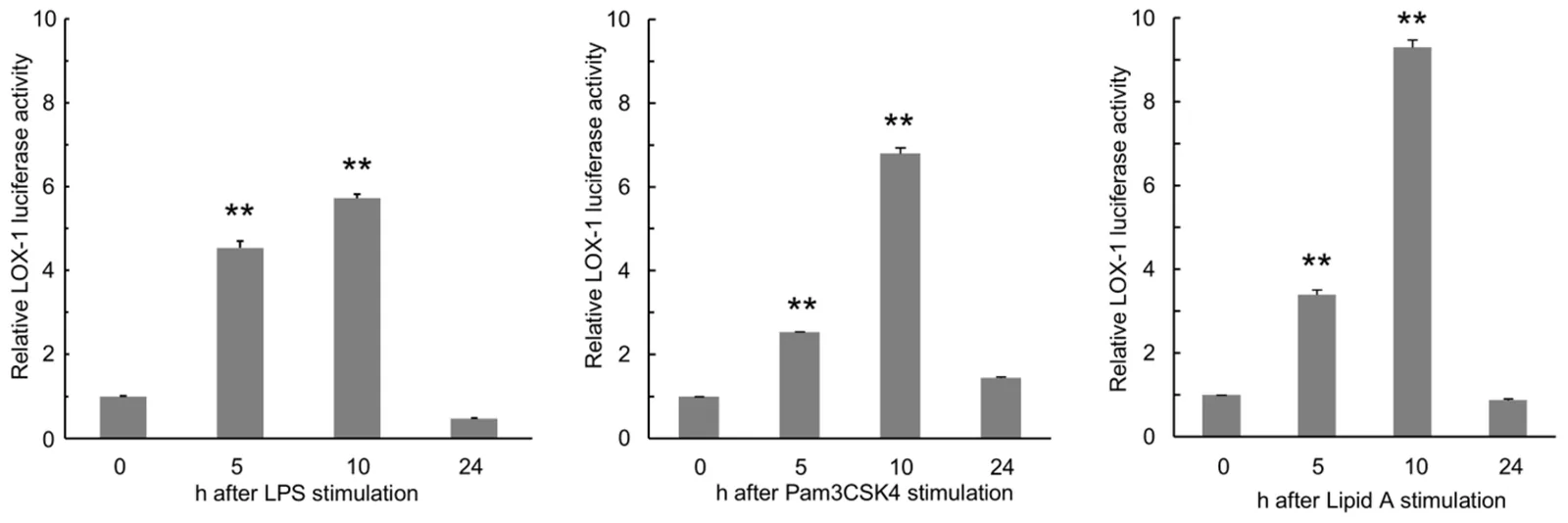
TLR2/4 stimulation transiently increases LOX-1 promoter activity in Raw264.7 cells. The murine LOX-1 promoter reporter construct was transfected into Raw264.7 cells. Cells were stimulated with *P. gingivalis* LPS, Pam3CSK4, or Lipid A for the indicated times. Luciferase activity was normalized to the corresponding untreated control (0 h) and expressed as relative promoter activity. Data are presented as mean ± SEM (*n* = 3). Statistical analysis was performed using one-way ANOVA followed by Dunnett’s post hoc test. ** *p* < 0.001.

To investigate whether TLR stimulation regulates LOX-1 transcriptional activity, LOX-1 promoter activity was assessed using a luciferase reporter assay in Raw264.7 cells. Cells were stimulated with *P. gingivalis* LPS, Pam3CSK4, or Lipid A. Luciferase activity significantly increased at 5 h and 10 h following stimulation with *P. gingivalis* LPS, Pam3CSK4, and Lipid A compared with baseline (0 h) (*p* < 0.001), indicating transient activation of the LOX-1 promoter. Promoter activity subsequently declined and returned to near-baseline levels by 24 h (Figure 1).

### 3.2. TLR2/4 Stimulation Regulates LOX-1 and MyD88 Expression in BMCs

To determine whether TLR2/4 stimulation regulates LOX-1 expression, BMCs were stimulated with Pam3CSK4 or Lipid A, and the expression levels of LOX-1 and MyD88 were analyzed by quantitative RT-PCR.

**Figure 2:**
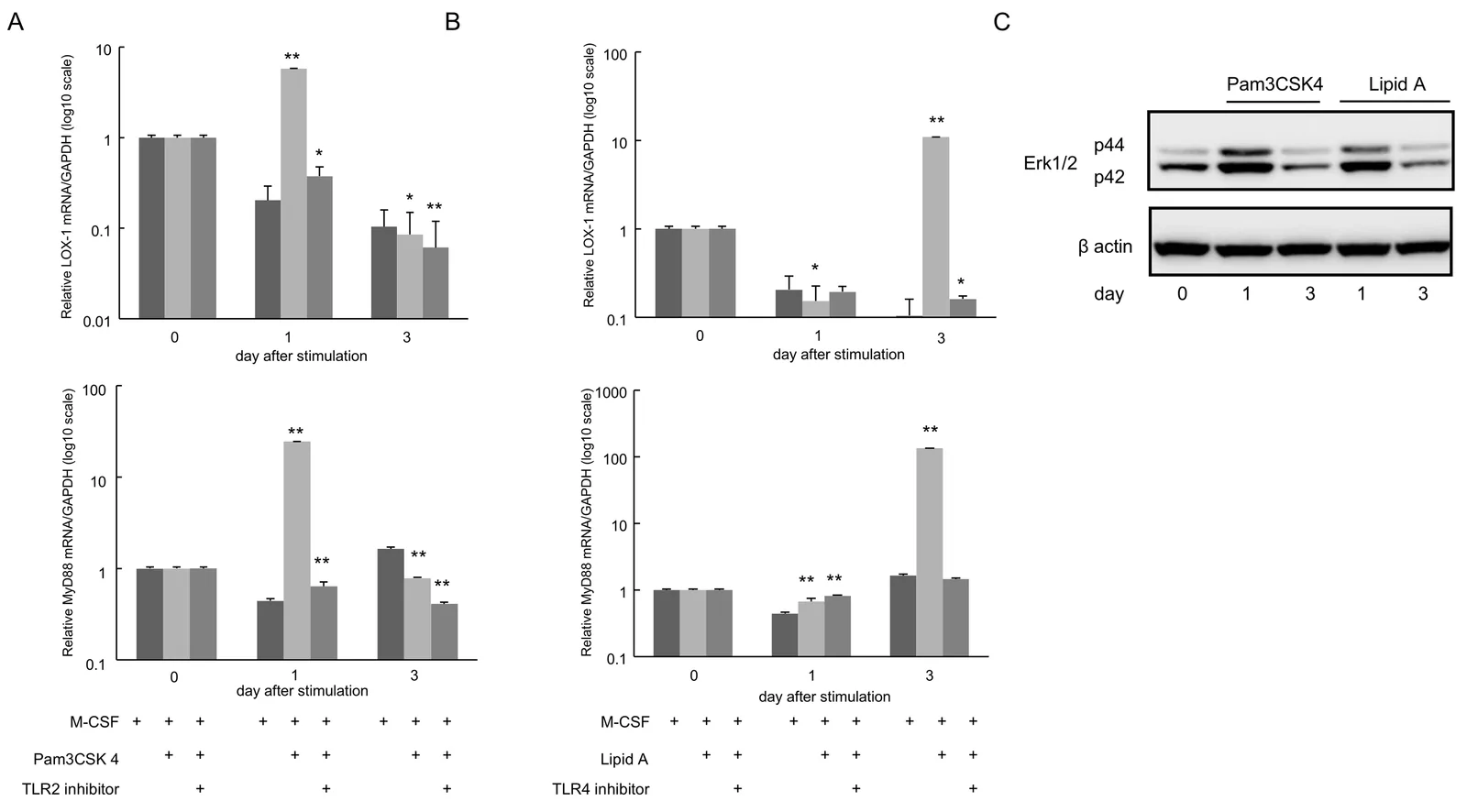

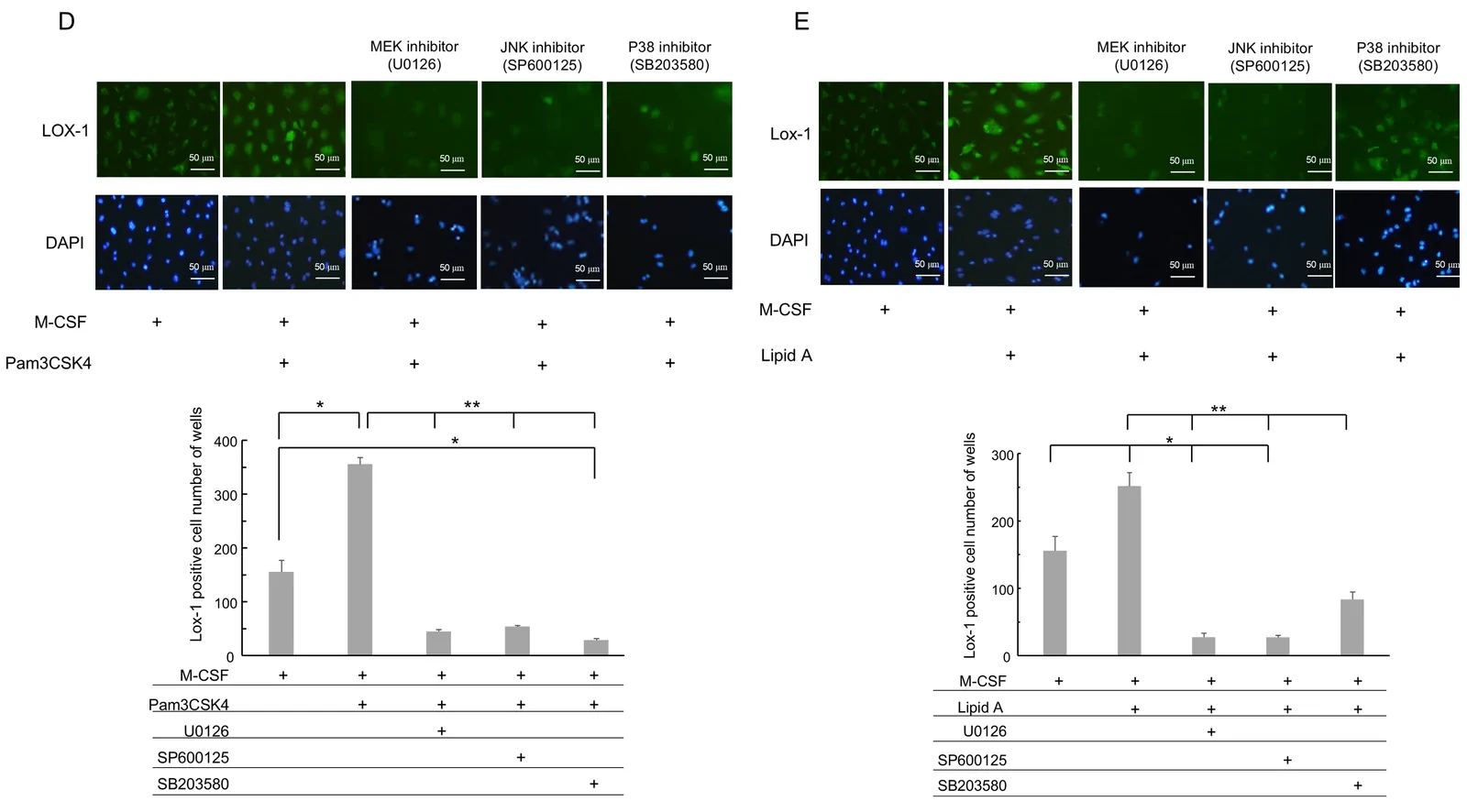
TLR2/4 stimulation regulates LOX-1 and MyD88 mRNA expression in BMCs. BMCs isolated from the tibiae and femora of mice and Raw264.7 cells were treated as described in Section 2 for the indicated times. Relative mRNA expression levels of LOX-1 and MyD88 in BMCs were normalized to GAPDH. Gene expression levels are presented as fold changes relative to the corresponding untreated controls and plotted on a log10 scale. Expression levels were analyzed on days 0, 1, and 3 following stimulation with Pam3CSK4 (**A**) or Lipid A (**B**). Western blot analysis demonstrated changes in ERK1/2 protein levels following Pam3CSK4 or Lipid A stimulation in Raw264.7 cells (**C**). The effect of U0126, SP600125 and SB203580 on TLR2/4-induced LOX-1 expression in BMCs were evaluates by immunofluorescence staining. Representative images of LOX-1 immunostaining (green) with DAPI nuclear counterstaining (blue) are shown, and the number of LOX-1-positive cells was quantified (**D**,**E**). Statistical analysis of gene expression data was performed using ΔCt values by one-way ANOVA followed by Dunnett’s post hoc test. Data are presented as the mean ± SEM (*n* = 3). * *p* < 0.05, ** *p* < 0.001. Cells were counted in four randomly selected microscopic fields per well, and the mean value per well was used for analysis (*n* = 4 wells per group). Welch’s ANOVA revealed significant differences among groups (* *p* < 0.05, ** *p* < 0.001) followed by Games–Howell post hoc testing (**D**,**E**).

Pam3CSK4 stimulation significantly increased LOX-1 and MyD88 mRNA expression on day 1, whereas expression levels decreased by day 3. These increases were significantly suppressed by treatment with a TLR2 inhibitor (Figure 2A). In contrast, Lipid A stimulation significantly increased both LOX-1 and MyD88 mRNA expression on day 3. The increase in LOX-1 expression was significantly inhibited by a TLR4 inhibitor (Figure 2B). ERK1/2 protein levels increased and were highest on day 1 following stimulation with both Pam3CSK4 and Lipid A (Figure 2C). Immunocytochemical analysis further confirmed increased LOX-1 expression following Pam3CSK4 and Lipid A stimulation. LOX-1 expression induced by Pam3CSK4 or Lipid A was significantly reduced by treatment with MAPK inhibitors (Figure 2D,E). Additional data are provided in Supplementary Figure S1.

### 3.3. oxLDL Cooperates with TLR Ligands to Promote Multinucleated TRAP-Positive Cell Formation in the Absence of Exogenous RANKL

To investigate the effects of oxLDL on osteoclastogenic responses including multinucleated TRAP-positive cell formation, spleen-derived BMMs were cultured with Pam3CSK4 or Lipid A in the presence or absence of oxLDL, and TRAP staining was performed.

**Figure 3:**
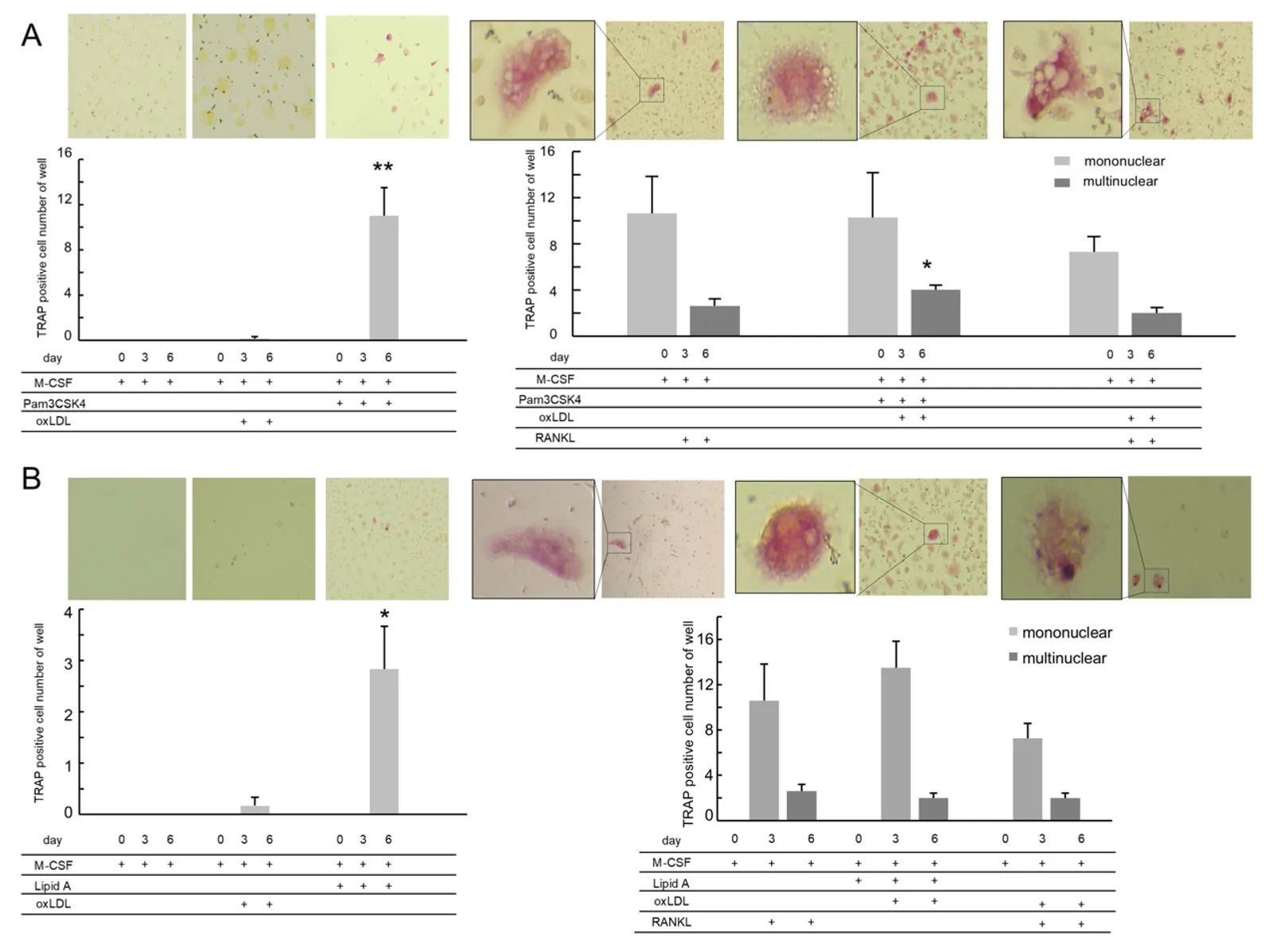
oxLDL cooperates with TLR ligands to promote multinucleated TRAP-positive cell formation in the absence of exogenous RANKL. Spleen-derived cells were cultured under the indicated conditions as described in Section 2. Treatment schedules are shown under each graph. Following stimulation with Pam3CSK4 (**A**) or Lipid A (**B**) in the presence or absence of oxLDL, cells were stained for tartrate-resistant acid phosphatase (TRAP). Representative higher-magnification images of the boxed regions  are shown to illustrate multinucleated TRAP-positive cells. The numbers of mononuclear and multinucleated TRAP-positive cells were quantified. Statistical analysis was performed using one-way ANOVA followed by Dunnett’s post hoc test. Data are presented as the mean ± SEM (*n* = 3). * *p* < 0.05, ** *p* < 0.001.

Stimulation with Pam3CSK4 or Lipid A alone significantly increased the number of mononuclear TRAP-positive cells but did not induce multinucleated TRAP-positive cell formation (Figure 3A, 3B). In contrast, co-stimulation with oxLDL and either PamCSK4 or Lipid A induced multinucleated TRAP-positive cells in the absence of exogenous RANKL. The numbers of multinucleated TRAP-positive cells were not significantly different from those induced by exogenous RANKL treatment. Representative higher-magnification images confirmed the presence of multinucleated TRAP-positive cells following co-stimulation with oxLDL and TLR ligands (Figure 3).

## 4. Discussion

In the present study, we investigated whether TLR2/4 stimulation regulates LOX-1 expression and how interactions between TLR signaling and oxLDL influence osteoclastogenic responses in vitro.

To determine whether TLR signaling regulates LOX-1 expression, we first examined the effects of representative TLR ligands on LOX-1 transcriptional activity. Stimulation with *P. gingivalis* LPS, Pam3CSK4, and Lipid A transiently increased LOX-1 promoter activity in Raw264.7 cells, suggesting that TLR signaling activates LOX-1 transcription. In BMCs, both Pam3CSK4 and Lipid A increased LOX-1 and MyD88 expression, and these effects were suppressed by TLR2 or TLR4 inhibitors. Collectively, these findings suggest that TLR-dependent signaling pathways associated with MyD88 expression contribute to LOX-1 induction [11,30].

A notable observation was that the temporal patterns of LOX-1 and MyD88 induction differed between TLR2 and TLR4 stimulation, with an earlier response to Pam3CSK4 and a delayed response to Lipid A. One possible explanation is that TLR4 activates both MyD88-dependent and TRIF-dependent signaling pathways, whereas TLR2 primarily signals through MyD88. Western blot analysis showed changes in ERK1/2 protein levels following TLR stimulation. Furthermore, pharmacological inhibition of ERK, JNK, and p38 MAPK attenuated TLR-induced LOX-1 expression, supporting the involvement of MAPK signaling. Although these pharmacological findings support the involvement of MAPK signaling, they do not establish causality, and further genetic loss-of-function studies are warranted. The additional involvement of TRIF-associated signaling may contribute to the distinct kinetics observed following TLR4 stimulation, although this possibility was not directly examined in the present study [35].

Overall, the present findings support the view that TLR activation is associated with increased LOX-1 expression, further extending previous observations describing functional interactions between these signaling pathways [30,34,36].

Osteoclasts are multinucleated bone-resorbing cells derived from monocyte/macrophage lineage precursors in response to RANKL and M-CSF stimulation [37,38]. Although TRAP activity is commonly used as a marker of osteoclast differentiation, TRAP-positive mononuclear cells are considered incompletely differentiated because they lack bone-resorbing activity despite expressing osteoclast-associated markers [39]. In the present study, oxLDL alone had minimal effects on TRAP-positive cell formation and did not induce multinucleated TRAP-positive cells. Similarly, stimulation with Pam3CSK4 or Lipid A alone failed to induce multinucleated TRAP-positive cells. Remarkably, co-stimulation with oxLDL and TLR ligands induced multinucleated TRAP-positive cell formation at levels comparable to those induced by RANKL, despite the absence of exogenous RANKL. However, because multinucleated TRAP-positive cell formation alone does not establish functional osteoclast differentiation, these findings should be interpreted as evidence of an osteoclastogenic response rather than definitive osteoclast differentiation. Taken together, the present observations indicate that inflammatory and metabolic signals act cooperatively to facilitate multinucleated TRAP-positive cell formation, although the underlying mechanism remains to be determined [11,40].

Periodontitis is a chronic inflammatory disease characterized by bacterial infection-associated alveolar bone destruction [1,2,3,4,5]. Because TLR2 and TLR4 recognize bacterial components derived from periodontal pathogens and initiate innate immune responses [6,7,8,9,10,11,12], the cooperative effects of TLR ligands and oxLDL observed in the present study may be relevant to inflammatory bone remodeling under dyslipidemic conditions.

Previous experimental studies have consistently shown that dyslipidemia and atherosclerosis are associated with altered bone metabolism and osteoporosis [41,42,43,44]. In addition, periodontal expression of TLR2 and TLR4 has been reported to increase in dyslipidemic animal models [45]. The present findings expand previous observations by demonstrating that oxLDL enhances cellular responses elicited by TLR stimulation under inflammatory conditions [30,34,45]. Collectively, the present observations indicate that dyslipidemia-associated oxLDL may amplify inflammatory osteoclastogenic responses triggered by bacterial TLR ligands, providing a potential mechanistic link between metabolic disorders and bone resorptive processes, including periodontitis.

Bone remodeling depends on coordinated activities of multiple cell types within the basic multicellular unit (BMU), including osteoclasts, osteoblasts, osteocytes, bone lining cells, osteomacs, and vascular endothelial cells, which coordinate bone resorption and bone formation to maintain skeletal homeostasis [46]. Since oxLDL has been reported to affect multiple cellular components within the BMU, dyslipidemia-associated inflammatory bone remodeling is likely to involve complex intercellular communication rather than effects on osteoclast precursors alone. Understanding these interactions may provide a broader perspective on the relationship between metabolic disorders and inflammatory bone diseases [47,48].

This study has several limitations. First, our experiments were performed in vitro using murine cells, and therefore the relevance of these findings to human periodontitis remains speculative. Additional studies using human cells, periodontal tissue-derived models, and in vivo models will be required to establish their translational significance. Second, multinucleated TRAP-positive cell formation alone does not fully establish functional osteoclast differentiation. Additional studies evaluating bone resorption activity and osteoclast-specific markers will be required to determine whether these cells possess mature osteoclast function. Third, although exogenous RANKL was not added, endogenous RANKL production by cells in the culture cannot be excluded. Therefore, the present findings demonstrate multinucleated TRAP-positive cell formation in the absence of exogenous RANKL rather than definitive RANKL-independent osteoclastogenesis. Fourth, further genetic loss-of-function studies targeting TLRs and LOX-1 will be necessary to establish the causal roles of these molecules in oxLDL- and TLR-mediated multinucleated TRAP-positive cell formation. Nevertheless, the present findings demonstrate that oxLDL cooperates with TLR signaling to promote multinucleated TRAP-positive cell formation under inflammatory conditions and improve our understanding of the interaction between metabolic and inflammatory pathways associated with osteoclastogenic responses. Future investigations will be needed to establish whether similar interactions occur in human osteoclast precursors and periodontal tissues. In addition, genetic loss-of-function studies targeting LOX-1 together with in vivo evaluation of osteoclast function will be important for establishing the pathological significance of the present findings.

In vivo studies have reported an association between elevated oxLDL levels and inflammatory alveolar bone loss in experimental periodontitis and dyslipidemic conditions [45]. Although the present study was performed in vitro, our findings provide a potential mechanistic basis for further investigation of how oxLDL may contribute to inflammatory bone remodeling under dyslipidemic conditions. These findings may also provide a framework for investigating the interaction between metabolic abnormalities and innate immune signaling in other inflammatory bone-resorptive diseases beyond periodontitis.

## 5. Conclusions

TLR2/4-mediated signaling may regulate LOX-1 expression and promote multinucleated TRAP-positive cell formation in cooperation with oxLDL, even in the absence of exogenous RANKL. oxLDL alone had minimal effects on TRAP-positive cell formation and was insufficient to induce multinucleated cell formation. In contrast, combined stimulation with TLR ligands and oxLDL induced multinucleated TRAP-positive cells at levels comparable to those induced by exogenous RANKL treatment. Collectively, our findings indicate that inflammatory and metabolic cues cooperate to facilitate multinucleated TRAP-positive cell formation under conditions in which exogenous RANKL is absent. Although further studies are needed to determine the functional properties of these cells and clarify the underlying signaling mechanisms, these findings provide a foundation for future studies investigating the interaction between dyslipidemia and inflammatory bone remodeling associated with periodontitis.

## Data Availability

The data supporting the present study can be obtained upon reasonable request.

## Supplementary Materials

The following supporting information can be downloaded at: https://www.mdpi.com/article/10.3390/medicina62081562/s1, Figure S1: Effects of anti-LOX-1 antibody on TLR ligand-mediated LOX-1 expression.

## Abbreviations

The following abbreviations are used in this manuscript:

oxLDL	Oxidized low-density lipoprotein
TLR	Toll-like receptor
LOX-1	lectin-like oxidized low-density lipoprotein receptor-1
RANKL	receptor activator of nuclear factor kappa-B ligand
BMC	bone marrow cell
BMM	bone marrow macrophage
M-CSF	macrophage colony-stimulating factor
LPS	lipopolysaccharide
TRAP	tartrate-resistant acid phosphatase
MyD88	myeloid differentiation factor 88
MAPK	mitogen-activated protein kinase
