# Supplementary material for: Oxidized Low-Density Lipoprotein Enhances Toll-like Receptor-Mediated Osteoclastogenic Responses in the Absence of Exogenous RANKL: Implications for Inflammatory Osteoclastogenesis in Periodontitis
Source: Medicina (Kaunas). 2026 Aug 14;62(8):1562. doi: 10.3390/medicina62081562 (PMC13515562; doi:10.3390/medicina62081562)
Supplement: Supplementary file 1 [file medicina-62-01562-s001.zip › supplementary 260802.pdf]

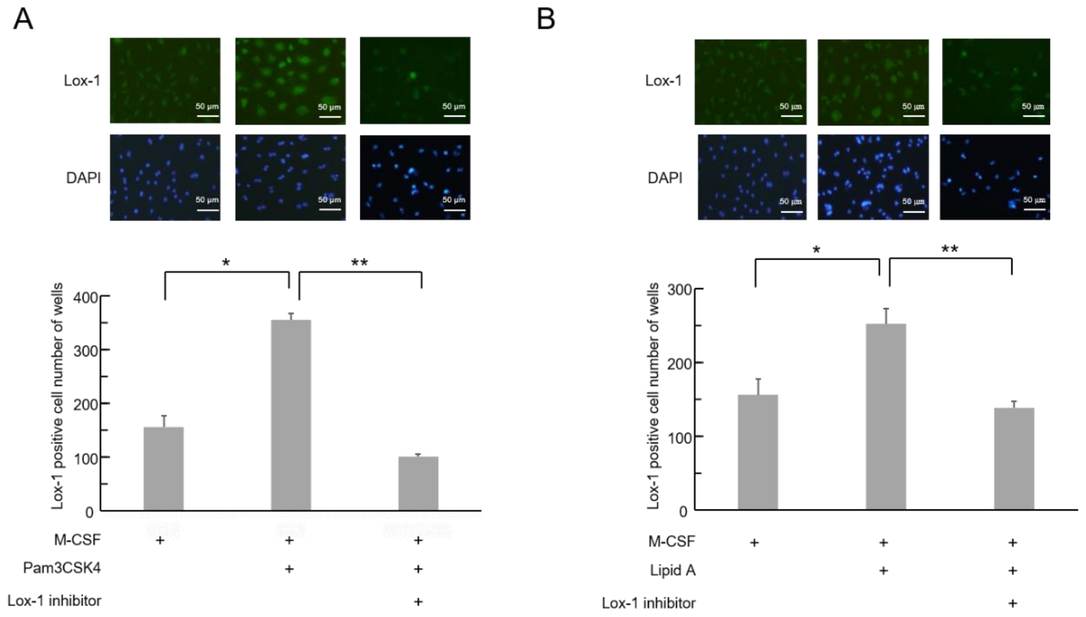

Supplementary Figure S1. Effects of anti-LOX-1 antibody on TLR ligand-induced Lox-1 expression.

BMCs isolated from the tibiae and femora of mice were treated as described in the Materials and Methods section for the indicated times. Effect of Lox-1 inhibitor on Pam3CSK4 (A) and Lipid A (B) induced Lox-1 expression by immunofluorescence staining. Representative images of Lox-1 immunostaining (green) with DAPI nuclear counterstaining (blue) are shown. The number of Lox-1-positive cells was quantified. Cells were counted in four randomly selected microscopic fields per well, and the mean value per well was used for analysis (n = 4 wells per group). Welch's ANOVA revealed significant differences among groups ($p < 0.001$), followed by Games–Howell post hoc testing.
